# Contribution of dynamic sentinel lymphoscintigraphy images to the diagnosis of patients with malignant skin neoplasms in the upper and lower extremities

**DOI:** 10.1186/2193-1801-3-625

**Published:** 2014-10-22

**Authors:** Hiroyuki Miura, Shuichi Ono, Koichi Shibutani, Hiroko Seino, Fumiyasu Tsushima, Shinya Kakehata, Katsumi Hirose, Hiromasa Fujita, Akihisa Kakuta, Masahiko Aoki, Yoshiomi Hatayma, Hideo Kawaguchi, Mariko Sato, Yoshihiro Takai, Takahide Kaneko, Daisuke Sawamura

**Affiliations:** Department of Radiology, Hirosaki University School of Medicine, 5 Zaifu-cho, Hirosaki, Aomori, 036-8562 Japan; Department of Dermatology, Hirosaki University School of Medicine, Hirosaki, Japan

**Keywords:** Sentinel lymphoscintigraphy, Dynamic image, Malignant skin neoplasm, Upper and lower extremities

## Abstract

The aim of the present study was to confirm the contribution of dynamic images in sentinel lymphoscintigraphy in malignant skin neoplasms: precisely, to investigate if dynamic images were necessary and to observe if dynamic images could reduce the areas needed for biopsy and dissection. Twenty-five patients with malignant skin neoplasms of the lower (n = 21) and upper (n = 4) extremities were retrospectively investigated. Images were evaluated by two independent reviewers, an expert in diagnostic radiology and nuclear medicine and a diagnostic radiologist in training. Visualized hot spots were assessed to be sentinel nodes using only static planar images. Next, both static planar and dynamic images were assessed. Reviewers scored diagnostic confidence values of determined sentinel nodes as follows: 0, cannot be decided; 1, possible; 2, probable; and 3, definitive. Patterns of lymphatic drainage were categorized into six different pathways: (1) inguinal type, (2) popliteal type, (3) inguinal and popliteal type, (4) axillary type, (5) cubital type, and (6) axillary and cubital type. In cases in the lower extremities, with dynamic images, the expert reviewer changed assessment in three cases and the trainee reviewer changed it in one case. There were no cases in which a decision was changed to be the same between both reviewers. Although the average diagnostic confidence value of assessment is usually higher with dynamic images, significant differences were not present. In cases of the upper extremities, both reviewers changed their assessment in one patient. By mutual agreement, cases in which assessment was changed with dynamic images were the inguinal and popliteal type, and the axillary and cubital type. The expert reviewer noticed lymphatic channels only visualized on dynamic images and changed assessment. Determination of whether or not a lymph node is a sentinel node depends on visualization of the lymphatic network. In the present circumstances, all biopsies of hot spots determined to be lymph nodes should not be excluded. However, excessive biopsies should be avoided as much as possible. It is necessary to use dynamic images alongside skillful observation.

## Introduction

Radioguided sentinel lymph node detection in malignant skin neoplasms is currently both common and indispensable (Gennari et al. 2000; Jacobs et al. 2001; Belhocine et al. 2002a; Alazraki et al. 2002; Rossi et al. 2006; Mariani et al. 2002; Mariani et al. 2004; Uren et al. 1996). In cases of malignant cutaneous neoplasms in the arms and legs, sentinel lymphoscintigraphy can occasionally reveal lymph nodes in the poples, cubitus, and several other unexpected areas (Uren et al. 2003; Vidal-Sicart et al. 2003; Vidal-Sicart et al. 2004; Tiffet et al. 2004; Matter et al. 2007) which are called interval nodes, in-transit lymph nodes, ectopic lymph nodes, and so on (Matter et al. 2007). The risk of metastasis to an interval node is similar to that of other sentinel lymph nodes, and the interval node may be the only metastatic site (Matter et al. 2007). Therefore, searching such areas should not be excluded in sentinel lymph node detection. Conversely, if all downstream lymph nodes in the groin and axilla are second echelon lymph nodes, interval node biopsy alone may be sufficient. This abbreviated lymph node dissection should reduce the invasiveness of the investigation. However, in some cases, there are lymphatic channels that are visualized only in early phase dynamic images immediately after tracer injection (Taylor et al. 1996; Maza et al. 2003; Toubert et al. 2008; Miura et al. 2010). If dynamic images are not observed, assessment of the sentinel lymph node might be different in these cases (Matter et al. 2007; Toubert et al. 2008; Miura et al. 2010). Therefore, it is important to determine if the hot spot is a sentinel lymph node, as this can be closely related to the patient’s prognosis (Gennari et al. 2000; Taylor et al. 1996; Maza et al. 2003). The aim of the present study was to confirm the contribution of dynamic images in sentinel lymphoscintigraphy: precisely, to investigate if dynamic images were necessary and to observe if dynamic images could reduce the areas needed for biopsy and lymph node dissection.

## Materials and methods

### Patients

Twenty-five patients with malignant skin neoplasms of the lower and upper extremities (10 male, 15 female; age range 51–84 years, mean age 68.6 years) were retrospectively investigated. Of the 25 patients, 21 had malignant cutaneous neoplasms in the upper extremities, and four had malignant cutaneous neoplasms in the lower extremities (Table 1). There were no cases with a prior history of serious external injury which could have brought about a change in lymphatic drainage. This sentinel lymph node detection by nuclear medicine was performed based on the guidelines of the Japanese Society of Nuclear Medicine and approved by the ethics committee of the in-hospital, and these patients were provided informed consent in writing.

**Table 1:** **Patient characteristics**

	Patient	Age (y)	Gender	Side	Primary tumor site	Pathology
Lower extremities	1	62	M	Right	Heel	Malignant Melanoma
	2	79	M	Right	Hallux	Malignant Melanoma
	3	57	F	Right	Hallux	Malignant Melanoma
	4	54	F	Left	Hallux	Malignant Melanoma
	5	78	F	Right	Planta pedis	Malignant Melanoma
	6	82	F	Left	Planta pedis	Malignant Melanoma
	7	72	F	Right	Planta pedis	Malignant Melanoma
	8	66	F	Left	Heel - planta pedis	Malignant Melanoma
	9	77	M	Left	Planta pedis	Malignant Melanoma
	10	76	F	Left	Heel	Malignant Melanoma
	11	50	M	Right	Planta pedis	Squamous cell carcinoma
	12	51	F	Left	Planta pedis	Malignant Melanoma
	13	57	F	Right	Hallux	Malignant Melanoma
	14	51	M	Left	Heel	Malignant Melanoma
	15	84	F	Right	Planta pedis	Malignant Melanoma
	16	80	F	Right	Planta pedis	Malignant Melanoma
	17	74	F	Left	Heel	Malignant Melanoma
	18	69	M	Right	Hallux	Malignant Melanoma
	19	84	M	Left	Heel	Malignant Melanoma
	20	74	F	Right	Heel	Malignant Melanoma
	21	72	M	Left	Planta pedis	Malignant Melanoma
Upper Extremities	22	53	M	Right	Thumb	Malignant Melanoma
	23	63	F	Left	Middle finger	Malignant Melanoma
	24	73	M	Right	Dorsum manus	Squamous cell carcinoma
	25	76	F	Right	Forefinger	Malignant Melanoma

### Imaging procedure

Approximately 150 MBq/0.5 mL of ^99m^Tc-phytate (FUJIFILM RI Pharma Co., Ltd., former Daiichi Radioisotope Laboratory Co., Ltd., Japan) was injected intracutaneously around the primary tumor or biopsy scar (Uren et al. 2003; Glass et al. 1998; Belhocine et al. 2002). Typically, four injections were required, although the number of injections depended on the size or location of the primary tumor or scar (Uren et al. 2003).

First, a dynamic scan of 10–20 min was aquired immediately after radiotracer injection (Pijpers et al. 1995; Murray et al. 2000). Next, anterior and posterior views of static scans were imaged approximately 20 min after tracer injection. At first, a dynamic scan of 10–20 min was performed immediately after radiotracer injection using a low-energy high-resolution (LEHR) collimator (MULTISPECT 2 gamma camera; Siemens, Germany) at one frame per 30 s for 30 frames with a 128 * 128 matrix. After the dynamic scan, about 20 min after tracer injection, static scans (anterior and posterior views, and oblique view if necessary) were performed for 3–5 min with a 512 * 512 matrix, using the LEHR collimator. Later, the gamma camera was renewed and a new imaging protocol was carried out as follows: a dynamic scan for 10–20 min was performed immediately after radiotracer injection using a LEHR collimator (Infinia Hawkeye gamma camera; GE Healthcare, USA) at one frame per 30 s, for 40 frames, with a 128 * 128 matrix. After the dynamic scan, about 20 min after tracer injection, static scans (anterior and posterior views, and oblique view if necessary) were performed for 3 min with a 512 * 512 matrix, using the LEHR collimator.

### Imaging observations

Obtained images were observed and evaluated by two independent reviewers. Reviewer A is an expert and certified as a specialist in diagnostic radiology and nuclear medicine. Reviewer B was training to be a specialist in diagnostic radiology. Final interpretation as determined by mutual agreement. Images were viewed on a liquid crystal display color monitor, and reviewers could freely change the window level and width.

### Assessment

**Figure 1:**
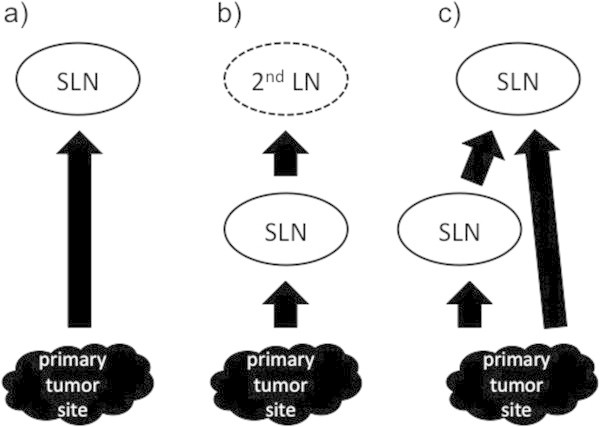
**Diagrams of the rules for the sentinel lymph node (SLN) assessment. a)** First hot spot thought to be a lymph node (LN) visualized along a lymphatic channel from the primary tumor site was considered a SLN, regardless of injection site. **b)** Second hot spot visualized along the same lymph channel was considered a second echelon lymph node. **c)** If the SLN and second echelon LN existed in the same site, both regarded as SLNs.

The first hot spot thought to be a lymph node visualized along a lymphatic channel from the primary tumor site was considered a sentinel lymph node, regardless of injection site. The second hot spot visualized along the same lymph channel was considered a second echelon lymph node (Miura et al. 2010). If the sentinel lymph node and second echelon lymph node existed in the same site, both were regarded as sentinel lymph nodes (Figure 1). Based on these rules, visualized hot spots were assessed to be sentinel lymph nodes by reviewers using only the static planar images. On another day, both static planar and dynamic images were assessed. Reviewers scored the diagnostic confidence value of determined sentinel lymph node as follows: 0, cannot be decided; 1, possible; 2, probable; and 3, definitive. Differences of diagnostic confidence values with or without dynamic images were confirmed by their average and Wilcoxson’s sign-rank sum test. In this statistical analysis, P-value < 0.05 was considered significant.

### Categorization of lymphatic flow

**Figure 2:**
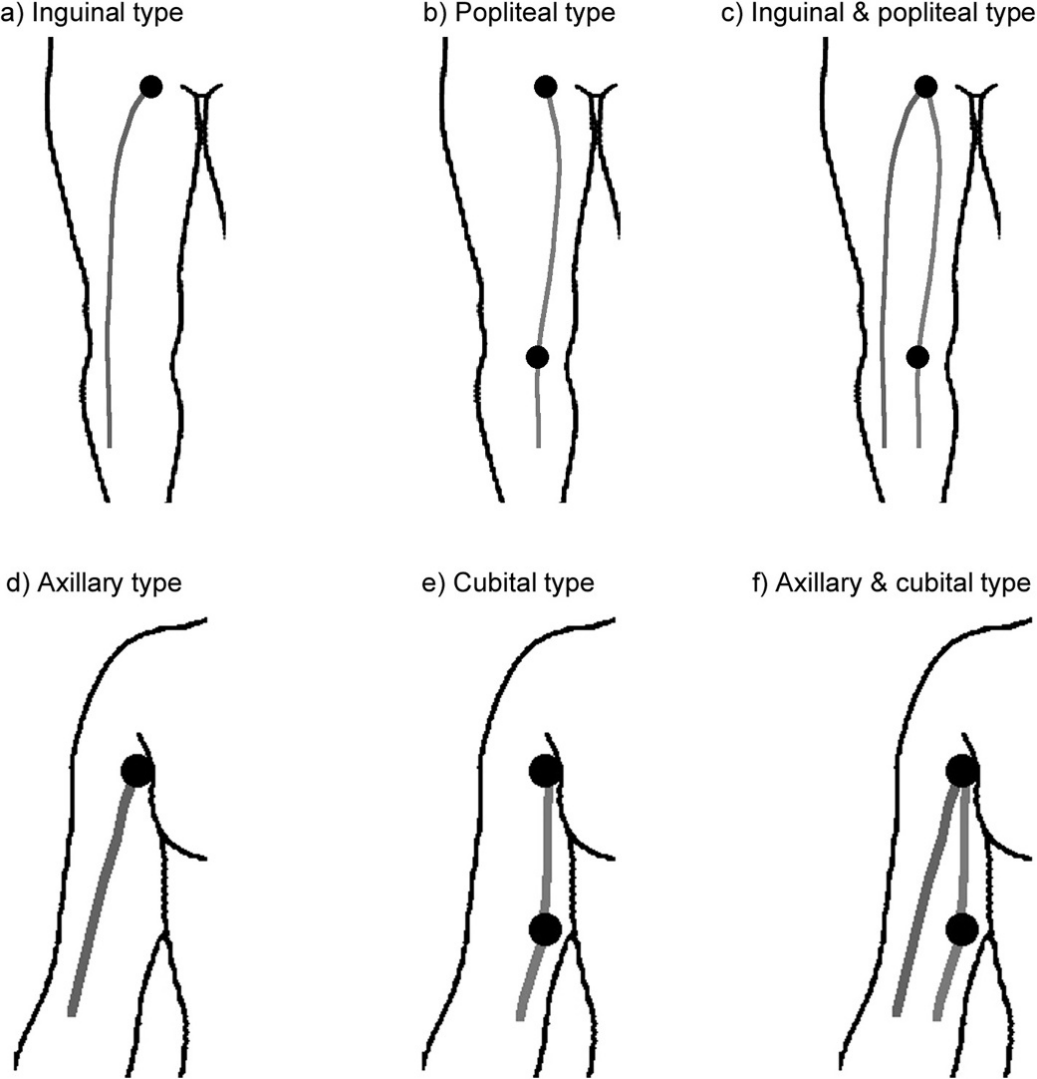
**Diagrams of the patterns of the categorized lymphatic drainage of the lower and upper extremities. a)** Inguinal type: primary tumor site to groin. **b)** Popliteal type: primary tumor site to poples. **c)** Inguinal and popliteal type: primary tumor site to groin and poples. **d)** Axillary type: primary site to axilla. **e)** Cubital type: primary site to cubitus. **f)** Axillary and cubital type: primary site to axilla and cubitus.

Patterns of lymphatic drainage from the skin of the lower extremities were categorized into three different pathways: (a) inguinal type, from the primary site to the groin (although there were plural lymphatic pathways in some cases, all paths drained into the groin, indicating that the sentinel lymph node was situated in the groin); (b) popliteal type, from the primary site to the poples; and (c) inguinal and popliteal type, from the primary site to the groin and from the primary site to the poples (Miura et al. 2010). Similarly, patterns of lymphatic drainage from the skin of the upper extremities were also categorized into three different pathways: (d) axillary type, from the primary site to the axilla; (e) cubital type, from the primary site to the cubitus; and (f) the axillary and cubital type, from the primary site to axilla and cubitus (Figure 2).

## Results

### Lower extremities

Reviewer A changed the sentinel lymph node assessment in three of the 21 cases with observation of dynamic images. Reviewer B changed the sentinel lymph node assessment in one of the 21 cases with observation of dynamic images. But there were no cases in which a decision was changed in the same way between reviewer A and B. Diagnostic confidence values with the observation of dynamic images scored by reviewer A were increased in five of 21 cases, and decreased in one case. Diagnostic confidence values with the observation of dynamic images scored by reviewer B were increased in four of 21 cases, and there were no decreased cases (Table 2). Average diagnostic confidence values with observation on dynamic images were higher than that without dynamic images except for cases in which popliteal lymph nodes were determined to be sentinel lymph nodes by reviewer A. However, significant differences as calculated by the Wilcoxson’s sign-rank sum test were not present (Table 3).

**Table 2:** **Sentinel lymph node assessment and diagnostic confidence values in cases in the lower extremities**

Patient	Reviewer A (expert radiologist)							Reviewer B (trainee radiologist)						
	Diagnostic confidence value						Assessment	Diagnostic confidence value						Assessment
	Only static			Static & dynamic				Only static			Static & dynamic			
	Groin	Poples	External iliac	Groin	Poples	External iliac		Groin	Poples	External iliac	Groin	Poples	External iliac	
1	1	2		2	3		I	1	3			3		C
2	3			3				3			3			
3	3			3		3	C	3			3			
4	3			3				3			3			
5	3			3				3			3			
6	1			3			I	2			2			
7	3			3		2	C	3			3			
8	3	3		3	3			1	3		3	3		I
9	3			3				3			3			
10	3			3				3			3			
11	3			3				3			3			
12	1	3		3	3		I	2	2		3	3		I
13	3			3				3			3			
14	3	3		3	3			2	3		3	3		I
15	3	3	2	3	3	3	I	3			3			
16	1	3		3	3		I		3			3		
17		3		3	3		C		3			3		
18	3			3				3			3			
19	3	3		3	1		D	3			3			
20	3			3				3			3			
21	3			3				2			3			I
Sentinel node sites	20/21	8/21	1/21	21/21	8/21	3/21		19/21	6/21	0/21	18/21	6/21	0/21	
%	95.2	38.1	4.8	100	38.1	14.3		90.5	28.6	0	85.7	28.6	0	

C: Assessment of sentinel lymph node was changed with observation of dynamic images.

I: Diagnostic confidence value increased with the observation of dynamic images.

D: Diagnostic confidence value declined with the observation of dynamic images.

**Table 3:** Average and P-values* of diagnostic confidence values in cases in the lower & upper extremities

		Cases in which inguinal nodes were determined to be sentinel node				Cases in which popliteal nodes were determined to be sentinel node			
		Reviewer A		Reviewer B		Reviewer A		Reviewer B	
		Only static	Static & dynamic	Only static	Static & dynamic	Only static	Static & dynamic	Only static	Static & dynamic
Lower extremities	Average	2.6	2.95	2.67	2.94	2.88	2.75	2.83	3
	*P*-value*	0.059		0.059		0.655		(Too few to calculate)	
Upper extremities	Average	2.5	3	2	3	3	3	3	3

*Wilcoxson’s sign-rank sum test.

### Upper extremities

Both reviewer A and B changed assessment of the sentinel lymph node in one patient (but not the same case). Diagnostic confidence values with observation of the dynamic images scored by both reviewer A and B were increased in one case (Table 4). Average diagnostic confidence values for cases in which axillary lymph nodes were determined to be the sentinel lymph node were increased with both reviewer A and B, but cases in which cubital lymph nodes were determined to be the sentinel lymph node were same in both reviewers. The number of cases was too small to calculate *P*-values with diagnostic confidence (Table 3).

**Table 4:** **Sentinel lymph node assessment and diagnostic confidence values in cases in the upper extremities**

Patient	Reviewer A (expert radiologist)							Reviewer B (trainee radiologist)						
	Diagnostic confidence value						Assessment	Diagnostic confidence value						Assessment
	Only static			Static & dynamic				Only static			Static & dynamic			
	Axilla	Cubitus	Brachium	Axilla	Cubitus	Brachium		Axilla	Cubitus	Brachium	Axilla	Cubitus	Brachium	
22	3			3				3			3			
23	2	3		3	3		I	1	3			3		C
24		3		3	3		C			1			3	I
25		3			3					3			3	
Number of sentinel sites	2/4	3/4	0/4	3/4	3/4	0/4		2/4	1/4	2/4	1/4	1/4	2/4	
%	50	75	0	75	75	0		50	25	50	25	25	50	

C: Assessment of sentinel lymph node was changed with observation of dynamic images.

I: Diagnostic confidence value increased with the observation of dynamic images.

### Interpretation by mutual agreement

In one in 21 cases in the lower extremities, the sentinel lymph node decision was changed with observation of dynamic images (an inguinal and popliteal type). On the other hand, two of four cases in the upper extremities, sentinel lymph node assessment was changed with observation of dynamic images (axillary and cubital type in both cases) (Table 5).

**Table 5:** **Assessment of SLN in the upper and lower extremities by mutual agreement**

A Lower extremities							B Upper extremities						
Patient	Assessment				Change	Category of lymphatic pattern*	Patient	Assessment				Change	Category of lymphatic pattern**
	Only static		Static & dynamic					Only static		Static & dynamic			
	Groin	Poples	Groin	Poples				Axilla	Cubitus	Axilla	Cubitus		
1	2nd	SLN	2nd	SLN	No	P	22	SLN		SLN		No	A
2	SLN		SLN		No	I	23	2nd	SLN	SLN	SLN	Yes	AC
3	SLN		SLN		No	I	24	2nd	SLN	SLN	SLN	Yes	AC
4	SLN		SLN		No	I	25	2nd	SLN	2nd	SLN	No	C
5	SLN		SLN		No	I	Number of SLN sites	2/4	3/4	2/4	3/4		
6	SLN		SLN		No	I	%	50	75	50	75		
7	SLN		SLN		No	I							
8	SLN	SLN	SLN	SLN	No	IP							
9	SLN		SLN		No	I							
10	SLN		SLN		No	I							
11	SLN		SLN		No	I							
12	SLN	SLN	SLN	SLN	Yes	IP							
13	SLN		SLN		No	I							
14	SLN	SLN	SLN	SLN	No	IP							
15	SLN		SLN		No	I							
16	SLN	SLN	SLN	SLN	No	IP							
17	2nd	SLN	SLN	SLN	Yes	IP							
18	SLN		SLN		No	I							
19	SLN	SLN	SLN	SLN	No	IP							
20	SLN		SLN		No	I							
21	SLN		SLN		No	I							
Number of SLN sites	17/21	7/21	20/21	7/21									
%	81	33.3	95.2	33.3									

SLN: sentinel lymph node.

2nd: second echelon lymph node.

*I: inguinal type.

*P: popliteal type.

*IP: inguinal and popliteal type.

**A: axillary type.

**C: cubital type.

**AC: axillary and cubital type.

**Figure 3:**
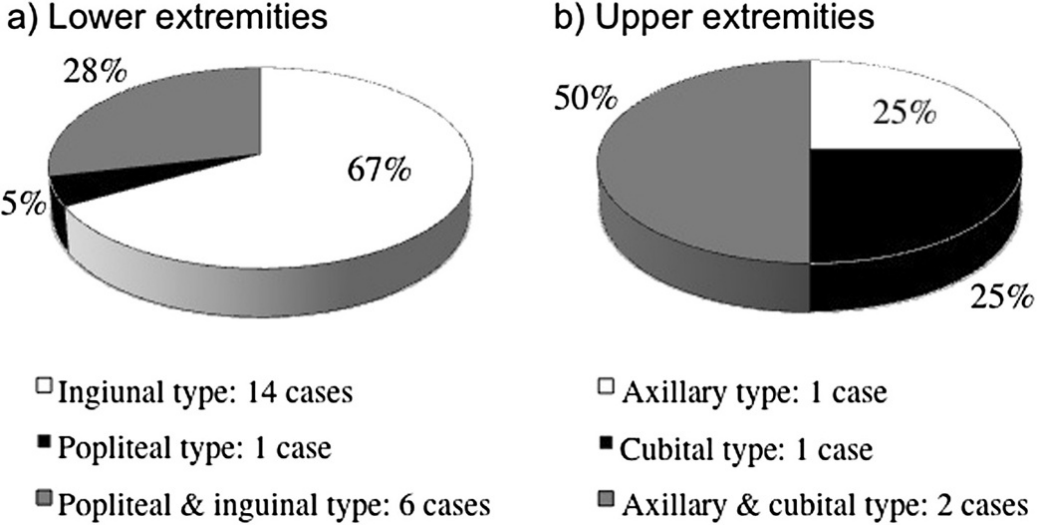
**Patterns of lymphatic drainage in cases of malignant skin neoplasms in the lower and upper extremities interpreted by mutual agreement. a)** In cases of the lower extremities: 14 inguinal type (67%), one popliteal type (5%), and 6 inguinal and popliteal type (28%). **b)** In cases of the upper extremities: one axillary type (25%), one cubital type (25%), and 2 axillary and cubital type (50%).

In cases in the lower extremities, lymphatic patterns were categorized as follows: 14 inguinal type (67%), one popliteal type (5%), and six inguinal and popliteal type (28%) (Miura et al. 2010). In cases in the upper extremities, they were categorized as follows: one axillary type (25%), one cubital type (25%), and two axillary and cubital types (50%) (Figure 3).

### Differentiation of sentinel lymph node determination with and without dynamic images

**Figure 4:**
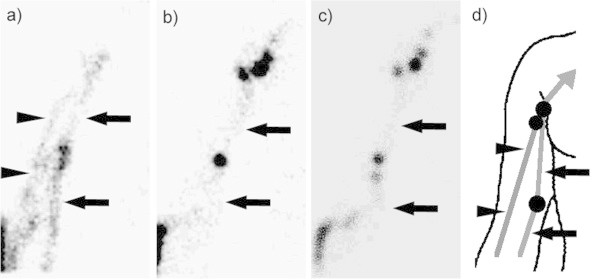
**A case of malignant melanoma of the right dorsum manus. (a)** Early-phase dynamic image (within 2 min of radiotracer injection) showed that lymphatic channels both passed through the cubitus (black arrow), and did not pass through the cubitus (black arrowheads). Therefore, both cubital and axillary LNs were diagnosed as SLNs. **(b)** Late-phase dynamic image (about 10 min after radiotracer injection) showed the lymphatic channel passing only through the cubitus (black arrow). **(c)** Static image about 20 min after tracer injection also showed the only lymphatic channel passing through the cubitus (black arrow). **(d)** Diagram of early-phase dynamic image. Without a dynamic scan, and without awareness of the lymphatic drainage only visualized on early-phase dynamic images, the axillary LN would have been incorrectly diagnosed as the second echelon LN.

Reviewer A (an expert radiologist) changed the sentinel lymph node assessment with observation of dynamic images in four of 25 cases. Reviewer A assessed the poples, cubitus, and groin (two cases) as sentinel lymph node sites with observation of static images only. They were changed in the following way with observation of static and dynamic images: groin to groin and poples, axilla to axilla and cubitus, and groin to groin and external iliac region. Concerning case No. 17, a lymphatic pathway without popliteal lymph node visualization was observed only on the early phase dynamic images. Similarly, with case No. 24, a lymphatic channel without cubital lymph node visualization was observed only on the early phase dynamic images. Reviewer A noticed these lymphatic channels with observation of dynamic images, and changed the sentinel lymph node assessment (Figure 4).

**Figure 5:**
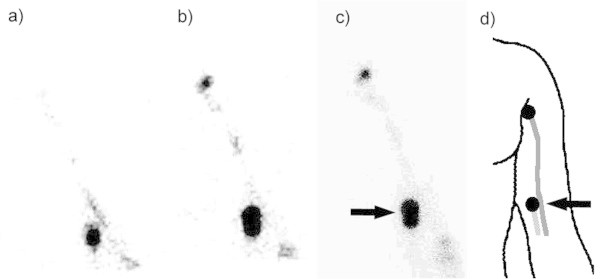
**A case of malignant melanoma of the left middle finger. (a)** Early-phase dynamic image showed a hot spot thought to be LN on the cubitus (black arrow). **(b)** Late-phase dynamic image showed the lymphatic channel to the axilla and a hot spot thought to be LN on the axillary region. **(c)** Static image about 20 min after tracer injection showed the hot spots on both cubitus (black arrow) and axilla, and lymphatic channels to the cubitus and axilla. **(d)** Diagram of late-phase dynamic image and static image. It is difficult to assess both lymphatic channels pass through the axillary LN (black arrow) or not because of blurred images.

On the other hand, reviewer B, a radiologist in training, changed sentinel lymph node assessment with observation of dynamic images in two of 25 cases. Reviewer B assessed the groin and poples and assessed the axilla and cubitus as sentinel lymph node sites with observation of static images only, and they were changed to poples only, and cubitus only, respectively. On these images, case No. 1 and No. 24, two lymphatic channels were close to the interval node, and reviewer B might have been uncertain as to if both lymphatic channels passed interval node or not (Figure 5). Interestingly, there were no cases in which an assessment was changed to reflect the same opinion of reviewer A and B.

### Discordance in sentinel lymph node assessment, reviewer A vs. reviewer B

1. Sentinel lymph node classification

Concerning cases No. 1, 16, and 17, reviewer A assessed the sentinel lymph nodes as being on the groin and poples (and external iliac), but reviewer B assessed the node on the poples only. Concerning cases No. 15 and 19, reviewer A assessed the sentinel lymph nodes as being on the groin and poples (and external iliac), but reviewer B assessed the nodes as being on the groin only. Concerning cases No. 23 and 24, reviewer A assessed the sentinel lymph nodes as being on the axilla and cubitus, but reviewer B assessed the nodes as being the cubitus or brachium.

2) Sentinel lymph node identification

Concerning cases No. 3 and 7, reviewer A identified the sentinel lymph nodes as being on the groin and external iliac region, but reviewer B identified the nodes as being on the groin only. Concerning cases No. 24 and 25, reviewer A identified the sentinel lymph nodes as being on the cubitus, but reviewer B identified the nodes as being on the brachium.

## Discussion

In different countries, various radiopharmaceuticals are used for radioguided sentinel LN detection (Glass et al. 1998). In Japan, mainly two radiopharmaceuticals, ^99m^Tc-labeled tin colloid and ^99m^Tc-labeled phytate, are the primary agents used in sentinel lymphoscintigraphy for malignant skin neoplasms (Higashi et al. 2003). Although ^99m^Tc-phytate is not a colloid, it combines with calcium in vivo and forms a colloid. The particle size of the phytate colloid varies according to the calcium concentration (Higashi et al. 2003), and its ranges from 200 to 1000 nm (Higashi et al. 2009). Therefore, dynamic scanning using ^99m^Tc-phytate makes it possible to visualize not only the SLNs but also the second echelon nodes and lymphatic channels (Miura et al. 2010).

A sentinel lymph node is defined as the first lymph node in a lymph node bed to receive lymphatic drainage from a tumor (Even-Sapir et al. 2003). A sentinel lymph node is not just the first node seen on dynamic imaging, because there may be multiple separate lymph channels that have different rates of lymph flow. If these channels drain to different nodes, then all of these nodes are sentinel lymph nodes, regardless of the time taken for the lymph containing the radiocolloid to reach them. A sentinel lymph node is also not necessarily the node closest to the primary site. Lymphatic vessels can bypass many nodes before reaching the sentinel lymph node (Uren et al. 2003). Therefore, sentinel lymph nodes are not always regional lymph nodes, and sentinel lymph nodes and second echelon lymph nodes may exist on the same site. Conversely, if it is possible to confirm interval node is sentinel lymph node and regional lymph node is not sentinel lymph node, interval node biopsy alone may be sufficient. However, assessment of sentinel lymph nodes of the upper and lower extremities was often different between the reviewers.

- Differences in sentinel lymph node assessment using dynamic images: difficulties in observation of dynamic images

Differences in sentinel lymph node assessment using dynamic images were decided by both reviewer A and B to exist between “inguinal & popliteal type” and “axillary & cubital type” nodes. In these cases, lymphatic channels existed that did not pass through the interval lymph node. In addition, lymphatic channels observed solely on early phase dynamic images. Therefore, if lymphatic channels that did not pass through the interval lymph node were visualized, even in a short period, lymph nodes into which both lymphatic channels flowed into were regarded as sentinel lymph nodes. Reviewer A, an expert diagnostic radiologist noted lymphatic channels visualized only by dynamic images, and changed the sentinel lymph node assessment. However, reviewer B, a trainee radiologist was not aware of the lymphatic channels visualized only by dynamic images, and did not change the sentinel lymph node assessment. These lymphatic channels were only observed during a short period (Miura et al. 2010; Glass et al. 1998), and were not clearly visualized because of faint radioactivity. In some cases, it is difficult to determine if lymphatic channels flow into lymph nodes or just overlap lymph nodes. However, clinical recurrence might also occur in interval nodes that are sentinel lymph nodes if they are not subjected to biopsy during initial surgical treatment, and interval nodes not be overlooked if the sentinel lymph node biopsy procedure is to be as accurate as possible in all patients (Uren et al. 2000). Therefore, careful observation with repetition and adjustment of window level and width is enormously needed.

b) Diagnostic confidence values of sentinel lymph node assessment of the lower extremities

In most cases, average diagnostic confidence values with observation of dynamic images for both reviewer A and B were higher than that without observation of dynamic images. However, significant differences were not present. In many cases, information about lymphatic flow was added with observation of dynamic images (Miura et al. 2010). Dynamic images were also useful when a hot spot could not be determined to be a lymph node, or a so-called “lymphatic lake”, that is, focal dilatation of lymphatic collecting vessel (Uren et al. 2003; Miura et al. 2010). However, too few upper extremities were analyzed in order to calculate diagnostic confidence value in the present study.

c) Categorization of visualized lymphatic patterns

In cases of the lower extremities, the “popliteal type” was found in one of 21 cases, only 5%. This fact may indicate that lymphatic patterns are limited in cases in which the popliteal lymph node is the sentinel lymph node, and all inguinal lymph nodes are second echelon lymph nodes (Miura et al. 2010; Kutsuna 1968).

In cases of the upper extremities, the “cubital type” was one of 4 cases, or 25%. There may possibly be many lymphatic patterns in which the cubital lymph node is the sentinel lymph node, and all axillary lymph nodes are second echelon lymph nodes. However, there were not enough cases of the upper extremities in the present study to conclude this.

d) Trustworthiness of dynamic images

There are many problems with dynamic images of sentinel lymphoscintigraphy. Because of the faint radioactivity of lymphatic vessels, visualization is often obscure: therefore, whether or not all lymphatic channels are visualized is questionable. Furthermore, lymphatic drainage often flows outside the image acquisition time. Overlapping lymphatic vessels often prevent comprehension of the correct lymphatic direction (Matter et al. 2007). Differentiation between a lymph node and a “lymphatic lake” (Uren et al. 2003; Matter et al. 2007) is sometimes difficult. Lymphatic lakes should not be mistaken for interval nodes, which retain tracer and are hot on delayed scans (Uren et al. 2003). Therefore, in the present circumstances, all biopsies of the hot spots determined to be lymph nodes should not be excluded in order to avoid the risk of undertreatment (Matter et al. 2007). However, excessive biopsies should be avoided as much as possible from the viewpoint of the sentinel lymph node concept (Pijpers et al. 1997). Many problems surrounding these issues remain to be resolved.

## Conclusions

The present study examined whether or not the use of dynamic images alters the outcome of sentinel lymph node assessment in patients with malignant skin neoplasms of the upper and lower extremities. Some lymphatic channels were observed using early phase dynamic images only, and sentinel lymph node assessment was altered in some cases. Determination of whether a lymph node is a sentinel lymph node depends on visualization of the lymphatic network. In the cases of the lower extremities, the average diagnostic confidence value of sentinel lymph node assessment is usually higher with observation of dynamic images. In many cases, information about lymphatic flow was added with observation of dynamic images and it was helpful for sentinel lymph node assessment. It is necessary to use dynamic images alongside skillful observation in assessment of sentinel lymph nodes.
